# Genome-wide identification of long noncoding RNA genes and their potential association with fecundity and virulence in rice brown planthopper, *Nilaparvata lugens*

**DOI:** 10.1186/s12864-015-1953-y

**Published:** 2015-10-05

**Authors:** Huamei Xiao, Zhuting Yuan, Dianhao Guo, Bofeng Hou, Chuanlin Yin, Wenqing Zhang, Fei Li

**Affiliations:** Department of Entomology, College of Plant protection, Nanjing Agricultural University, Nanjing, 210095 China; Department of City Construction, Shaoyang University, Shaoyang, 422000 China; State Key Laboratory for Biocontrol/Institute of Entomology, Sun Yat Sen University, Guangzhou, 510275 China; Ministry of Agriculture Key Lab of Agricultural Entomology, Institute of Insect Sciences, Zhejiang University, 866 Yuhangtang Road, Hangzhou, 310058 China

**Keywords:** Long noncoding RNAs, RNA-seq, Genome location, Co-expression, Fecundity, Virulence

## Abstract

**Background:**

The functional repertoire of long noncoding RNA (lncRNA) has been characterized in several model organisms, demonstrating that lncRNA plays important roles in fundamental biological processes. However, they remain largely unidentified in most species. Understanding the characteristics and functions of lncRNA in insects would be useful for insect resources utilization and sustainable pest control.

**Methods:**

A computational pipeline was developed to identify lncRNA genes in the rice brown planthopper, *Nilaparvata lugens*, a destructive rice pest causing huge yield losses. Strand specific RT-PCR were used to determine the transcription orientation of lncRNAs.

**Results:**

In total, 2,439 lncRNA transcripts corresponding to 1,882 loci were detected from 12 whole transcriptomes (RNA-seq) datasets, including samples from high fecundity (HFP), low fecundity (LFP), I87i and C89i populations, in addition Mudgo and TN1 virulence strains. The identified *N. lugens* lncRNAs had low sequence similarities with other known lncRNAs. However, their structural features were similar with mammalian counterparts. *N. lugens* lncRNAs had shorter transcripts than protein-coding genes due to the lower exon number though their exons and introns were longer. Only 19.9% of *N. lugens* lncRNAs had multiple alternatively spliced isoforms. We observed biases in the genome location of *N. lugens* lncRNAs. More than 30% of the lncRNAs overlapped with known protein-coding genes. These lncRNAs tend to be co-expressed with their neighboring genes (Pearson correlation, *p* < 0.01, T-test) and might interact with adjacent protein-coding genes. In total, 19-148 lncRNAs were specifically-expressed in the samples of HFP, LFP, Mudgo, TN1, I87i and C89i populations. Three lncRNAs specifically expressed in HFP and LFP populations overlapped with reproductive-associated genes.

**Discussion:**

The structural features of *N. lugens* lncRNAs are similar to mammalian counterparts. Coexpression and function analysis suggeste that *N. lugens* lncRNAs might have important functions in high fecundity and virulence adaptability.

**Conclusions:**

This study provided the first catalog of lncRNA genes in rice brown planthopper. Gene expression and genome location analysis indicated that lncRNAs might play important roles in high fecundity and virulence adaptation in *N. lugens*.

**Electronic supplementary material:**

The online version of this article (doi:10.1186/s12864-015-1953-y) contains supplementary material, which is available to authorized users.

## Background

The development of high-throughput techniques has accelerated the sequencing of insect genomes and transcriptomes, leading to the rapid accumulation of insect gene data. Currently, 156 insect genomes have been sequenced and were deposited in the NCBI genome database [1], mainly from Diptera, Lepidoptera, and Hymenoptera. Hundreds of insect transcriptomes have been submitted to the NCBI SRA database [2]. Huge amounts of insect RNA-seq data provide valuable resources to retrieve gene sequences and to estimate gene abundance by counting the read numbers [3]. However, major works on insect genome annotation and RNA-seq analysis have been limited to protein-coding genes.

Increasing evidence has showed that noncoding RNA (ncRNA) genes exist widely in the genomes of almost all organisms [4, 5]. ncRNAs are arbitrary classified into two types based on their sizes. One type is small RNAs, which are shorter than 200 nucleotides (nt), including but not limited to microRNAs (miRNAs), Piwi-interacting RNAs (piRNAs), small nucleolar RNAs (snoRNAs), and transfer RNAs (tRNAs). The other type is long noncoding RNAs (lncRNAs), with transcripts longer than 200 nt that lack protein-coding potential [6]. The lncRNAs located in the intergenic region are named as long intergenic noncoding RNAs (lincRNAs). LncRNAs with transcripts longer than 50 Kb are defined as very long noncoding RNAs (vlncRNAs) [7]. RNA-sequencing (RNA-seq) data are very useful resources to identify lncRNAs. Several international genome consortia, such as FANTOM, ENCODE, GETx, and modENCODE, have developed several computational approaches and identified thousands of lncRNA genes from a variety of species [8–12]. More than 9000 lincRNA genes were discovered in the human genome [8, 13–17] and >10,000 lincRNAs were found in the mouse genome. By analyzing 93 samples and expressed sequence datasets, 6621 lincRNAs from 4515 gene loci were identified from the pig genome [18]. In a chicken RNA-Seq dataset, Li et al. found 281 novel lincRNA genes associated with muscle development [10]. Jenkins et al. used a computational pipeline to identify lncRNAs from multiple *Anopheles gambiae* deep RNA-seq data, yielding 2949 lncRNA genes. These lncRNAs showed differential expression across the life stages. The secondary structures of lncRNAs are highly conserved within the Gambiae complex [19]. As an important model organism, *Drosophila melanogaster* has been extensively investigated for its lncRNA genes. Several efforts have identified 3193 lncRNA genes in *D. melanogaster* [20–22].

Distinct roles have been characterized for only a small subset of lncRNAs and the function of the vast majority of lncRNAs remains unknown. Several studies have shown that lncRNAs play essential roles in a wide variety of fundamental biological processes, such as cell differentiation [16], pluripotency maintenance [23], transcription regulation [24], epigenetic regulation [25, 26], dosage compensation [27], and tumorigenesis [16]. In *D. melanogaster,* a yellow-achaete intergenic RNA (yar) affects sleep behavior. *Yar* is conserved in *Drosophila* species [28]. A neural-specific lncRNA, CRG, regulates the locomotor activity and climbing ability in *Drosophila* [29]. These studies suggested that lncRNAs have much more important roles than expected.

The rice brown planthopper, *N. lugens*, is one of the most destructive insect pests in rice production. It directly sucks the phloem sap and transmits viruses, causing huge yield losses. The rice brown planthopper has two types of wings, long wing and short wing. The wing dimorphism is regulated by insulin receptors [30]. The long-winged brown planthopper migrates from tropical to temperate regions in summer and then back to the tropics in the autumn. In the immigrant areas, the brown planthopper population increases very quickly in one or two generations. This notorious pest has repeatedly adapted to resistant rice varieties used for pest control [31]. The high fecundity and virulence adaptation of *N. lugens* are major factors causing the high damage to rice. Insecticides are one of the most widely used methods to control rice brown planthopper. However, overuse of insecticides has resulted in resistance, resurgence, and residues. Understanding the mechanism of high fecundity and virulence adaptation is important to develop alternative pest control strategies. Here, we constructed a computational pipeline to identify lncRNAs from RNA-seq datasets of 12 samples of rice brown planthopper. We identified several lncRNAs specifically expressed in a high fecundity *N. lugens* population and found that expression patterns of lncRNAs varied between *N. lugens* strains/populations, suggesting that lncRNAs might have key roles in the fecundity and virulence of the rice brown planthopper.

## Results

### Identification and validation of lncRNAs in *N. lugens*

**Fig. 1:**
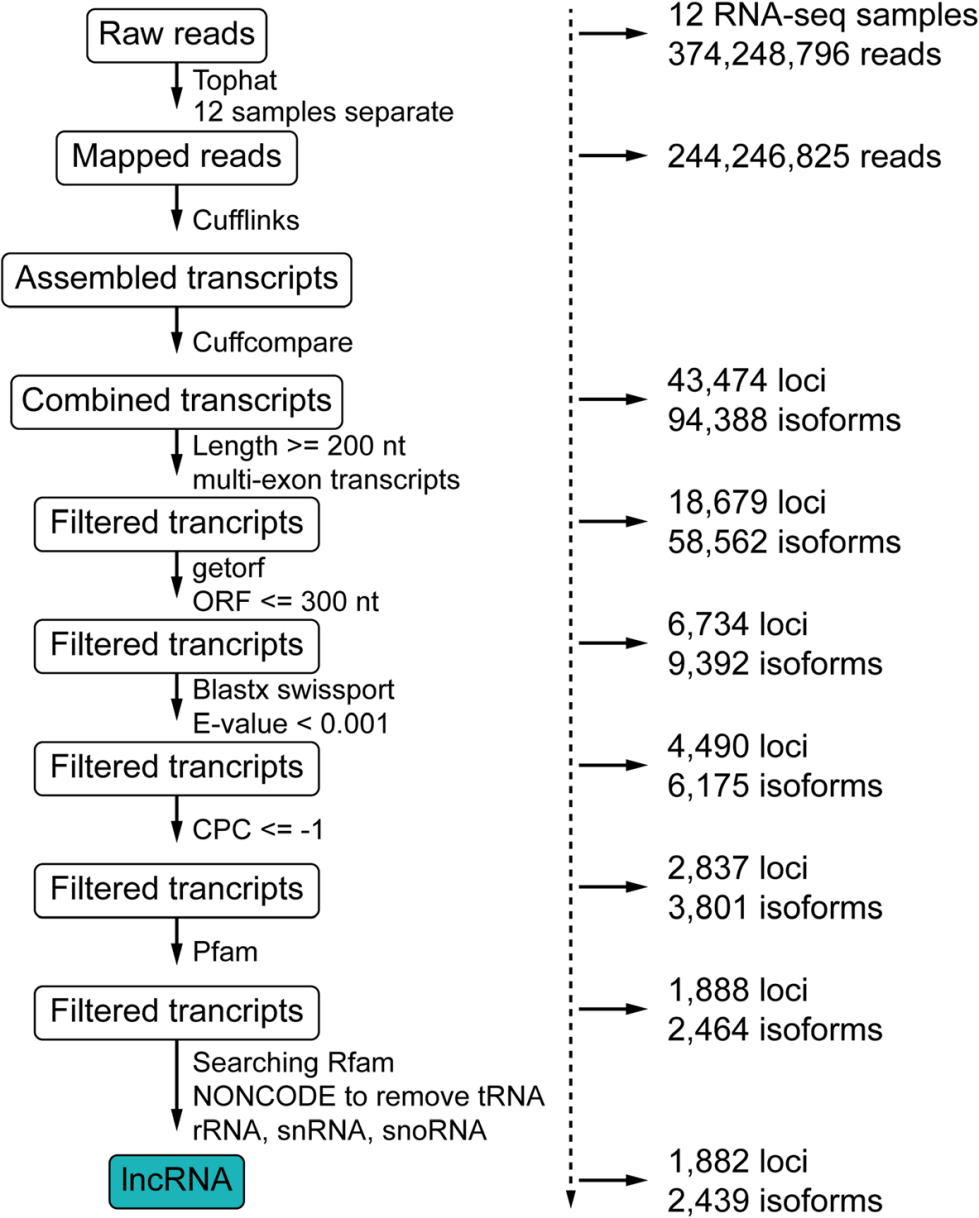
The computational pipeline for identifying lncRNA genes in *N. lugens* from RNA-seq data

A computational pipeline was developed to identify lncRNA genes from the *N. lugens* transcriptome (Fig. 1). This pipeline was applied on 12 different *N. lugens* transcriptome datasets and yielded 2439 transcript isoforms corresponding to 1882 loci from 12 *N. lugens* RNA-seq datasets (Additional file 1: Text file containing identified lncRNA sequences). According to genome location, we divided these lncRNA transcripts into seven types according to the guide of the HUGO Gene Nomenclature Committee (HGNC) [7]. Intergenic lncRNA transcript were named as BPHLINCxxx (xxx means number). Intronic lncRNAs, which occurred entirely within an intron were named as BPHOGSxxx-IT. BPHOGS is the official gene set of protein coding genes. LncRNAs that overlapped with a reference intron or exon on the opposite strand were named as BPHOGSxxx-AS. LncRNAs that overlapped with a reference exon or splice junctions on the same strand were named as BPHOGSxxx-OT. Those lncRNA that could not be classified as any of the above types and were regarded as unclassified and were named as BPHLNC-unc.

The individual datasets were analyzed separately using the computational pipeline. In total, 948 and 1562 lncRNA genes were found in the 2-day-old adults of the LFP and HFP populations, respectively, whereas 1324 and 1563 lncRNAs were identified in the fifth instar larvae of the LFP and HFP populations. A higher number of lncRNAs (1798–2081) were discovered in the fat body, salivary gland, and antennae of the virulence-associated Mudgo and TN1 populations. 1618, 1806, and 1721 lncRNAs were found in the wild, I87i, and C89i populations, respectively (Table 1). There are several factors influencing the numbers of identified lncRNAs in the different samples. The sequencing coverage is one of the major factors. In general, the higher the coverage, the more lncRNAs could be identified (Additional file 2: Figure S1). However, there were some exceptions. The sample of 2-days-old adults of the HFP population had the lowest coverage, but 1562 lncRNA genes were identified in this sample, suggesting that lncRNA genes might have important roles in high fecundity. By contrast, the wild and I87i population had the highest coverage but they did not contain the most lncRNAs.

**Table 1:** The numbers of lncRNAs in individual RNA-seq datasets of different *N. lugens* strains/populations and in the comprehensive RNA-seq dataset

lncRNA types^a^	lncRNA in 12 samples		Fecundity^d^				Virulence					Populations		
	Number of lncRNA	Percentage (%)	LFP^b^ adult 2 d	HFP^b^ adult 2 d	LFP 5^th^ instar larva	HFP 5^th^ instar larva	Mudgo^c^ fat body	TN1^c^ fat body	Mudgo salivary gland	TN1 salivary gland	TN1 antenna	I87i^c^ population	C89i^c^ population	Wild population
Intergenic^1^	853	34.97	344	534	440	536	694	682	739	727	569	603	560	496
Intronic^2^	80	3.28	30	42	38	41	57	56	70	67	55	57	49	53
Intronic overlap^3^ (−)	5	0.21	1	3	0	1	4	4	5	5	2	4	4	3
Exonic overlap^4^ (+)	385	15.79	151	250	215	247	311	310	323	332	305	287	271	255
Exonic overlap^5^ (−)	211	8.65	78	137	117	147	175	176	188	187	153	147	145	139
Splice junction overlap^6^	264	10.28	103	172	150	175	218	221	227	226	199	197	190	188
Unclassified^7^	641	26.28	241	424	364	416	528	526	559	537	515	511	502	484
total	2,439	100	948	1,562	1,324	1,563	1,987	1,975	2,111	2,081	1,798	1,806	1,721	1,618

^a^LncRNA types: 1) Intergenic transcript, 2) falling entirely within a reference intron, 3) overlaps with a reference intron on the opposite strand, 4) overlaps with a reference exon, 5) overlaps with a reference exon on the opposite strand, 6) At least one splice junction is shared with a reference transcript, 7) Unclassified

^b^ *LFP* low fecundity population, *HFP* high fecundity population

^c^ *TN1*: avirulent Taichung Native 1 host strain, *Mudgo*: virulent (carrying the resistance gene bph1) host strain, *I87i* Izumo87 strain, *C89i*: Chikugo89 strain

^d^Based on the analysis of the 12 RNA-seq datasets together using the computational pipeline developed

**Fig. 2:**
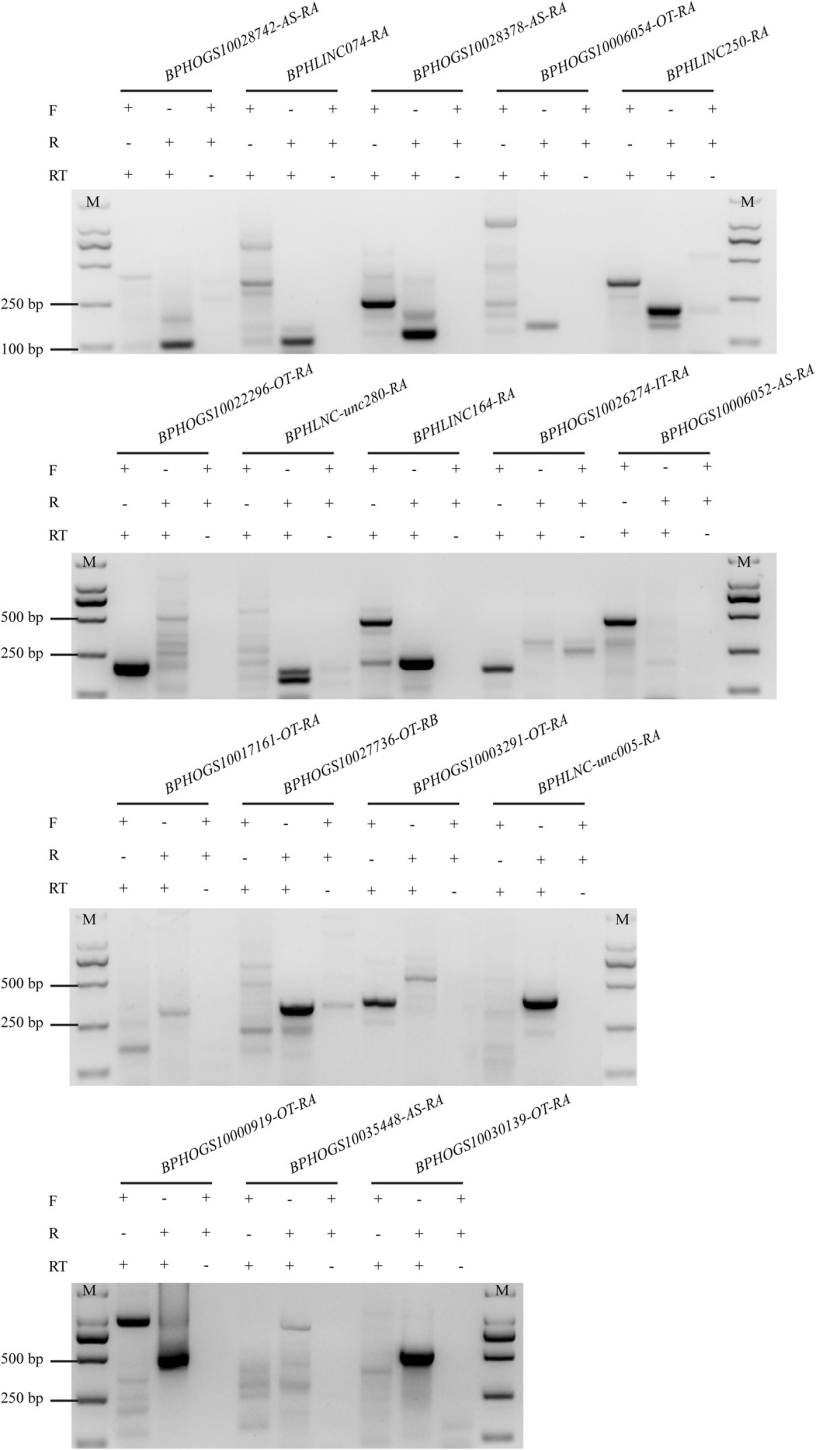
Strand-specific PCR of 17 randomly selected lncRNAs to determine the transcription orientation. BPHOGS10035448-AS-RA was not amplified with a correct band. So, 16 lncRNAs were successfully amplified and confirmed by sequencing (see Figure S2 for RT-PCR validation). The results indicated that 12 lncRNA transcribed from the antisense strand, and four from the sense strand. F: forward primer (sense); R: reverse primer (antisense); RT: reverse transcriptase

To confirm the reliability of the identified lncRNA genes, we selected 20 lncRNAs for RT-PCR validation. seventeen lncRNAs were successfully amplified (Additional file 3: Figure S2), suggesting that a high percentage of lncRNAs detected by this pipeline were reliable in terms of expression. The transcription orientation of these 17 lncRNAs were determined by strand-specific RT-PCR. Sixteen out of them were successfully amplified. The results demonstrated that four lncRNAs were transcribed from the sense strand whereas 12 lncRNAs from the antisense strand (Fig. 2).

### Structural features of lncRNAs in *N. lugens*

**Fig. 3:**
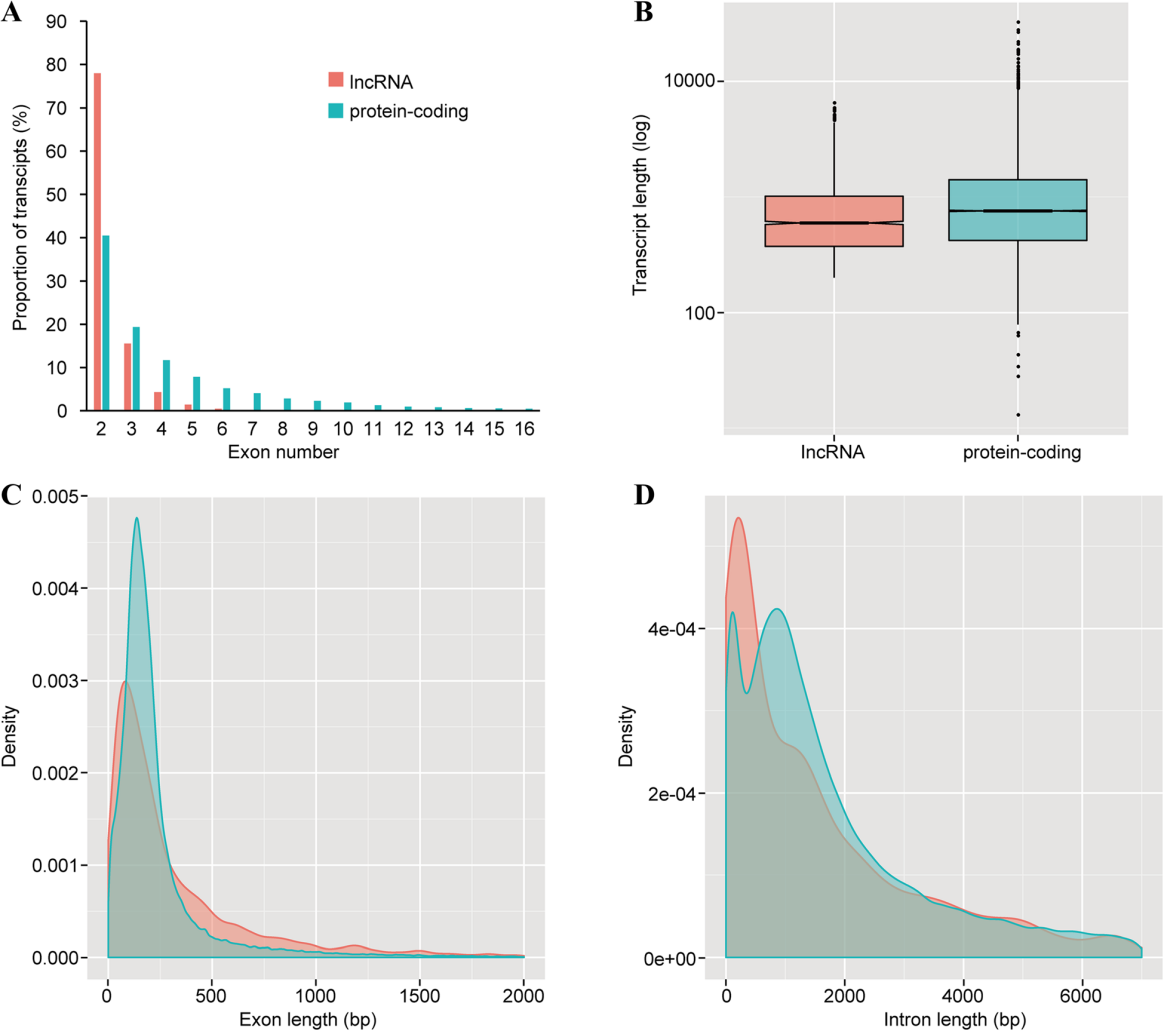
Structural gene features of *N. lugens* lncRNA genes. **a** The number of exons per transcript of lncRNAs and protein-coding genes. The majority of lncRNAs have only two exons. **b** The transcript lengths of lncRNA and protein-coding gene. On average, lncRNAs have short transcripts. **c** The distribution of exon sizes of lncRNA and protein-coding genes. **d** The distribution of intron sizes of lncRNA and protein-coding genes. The density in Y-axis means the area under the curve of a density function represents the probability of getting an x value between a range of x values. *Red*: lncRNA, *green*: protein-coding genes

We analyzed the structural features of lncRNA genes in *N. lugens*. Consistent with their counterparts in the mammals, *N. lugens* lncRNAs had fewer exons than protein-coding genes (Fig. 3a). 77.9 % of *N. lugens* lncRNAs had only two exons, which is nearly twice the ratio of 40.4 % observed in protein-coding genes. By contrast, only 4.34 % of lncRNA genes had four exons, which is significantly lower than 11.70 % of protein-coding genes. The average transcript length of *N. lugens* lncRNAs was 841 bp whereas that of protein-coding genes was 1106 bp (Fig. 3b). Interestingly, lncRNA genes had longer exons (363 bp on average) and longer introns (7792 bp on average) than protein-coding genes (250 bp exons and 2583 bp introns, Fig. 3c and d). However, lncRNAs had shorter transcripts than protein-coding genes because of the lower number of exons.

**Fig. 4:**
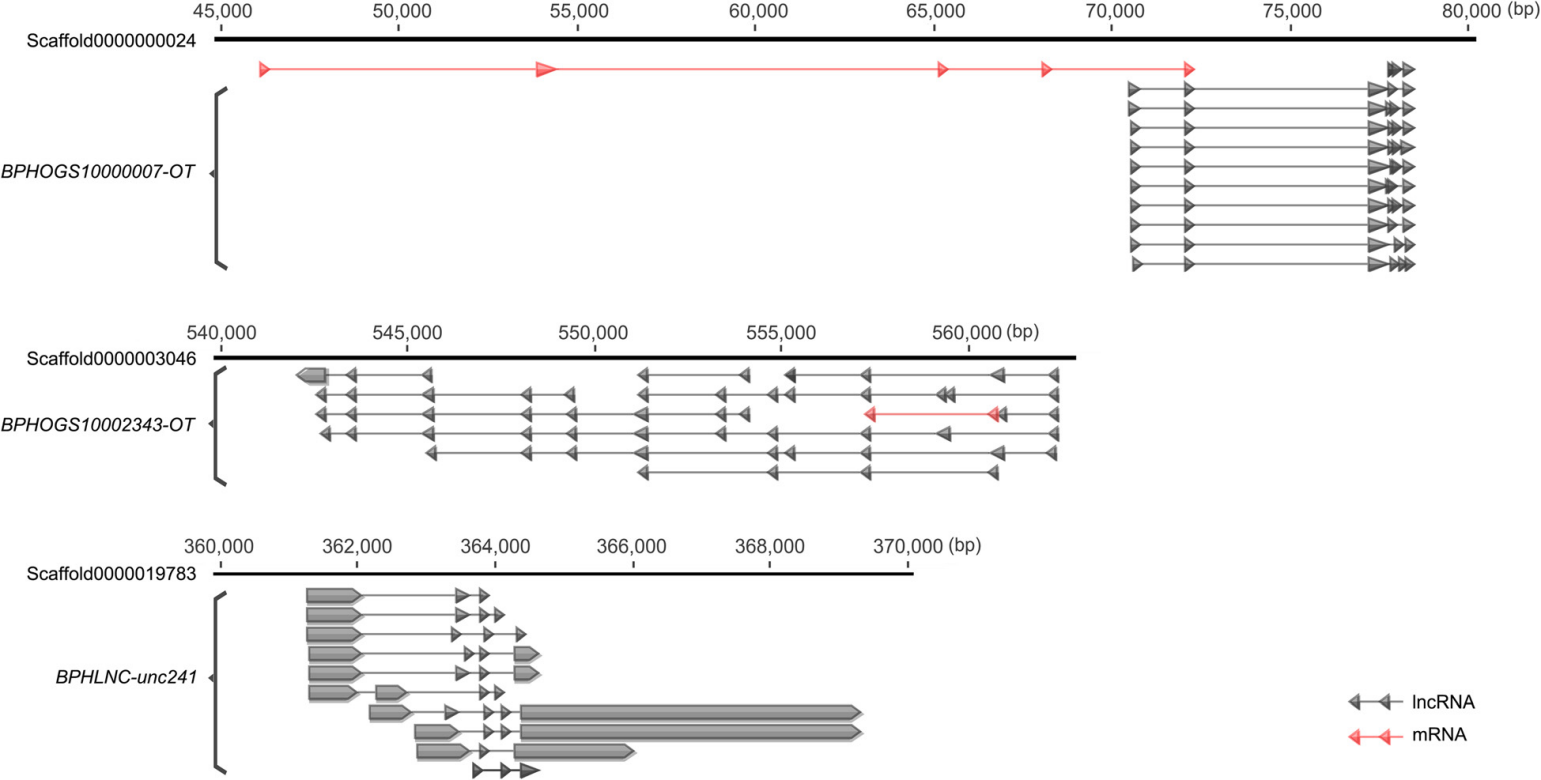
Gene structures of three lncRNA genes that had the most alternatively spliced isoforms. *BPHOGS10000007-OT* and *BPHOGS10002343-OT* have 11 spliced isoforms and *BPHLNC-unc241* has 10 spliced isoforms

**Fig. 5:**
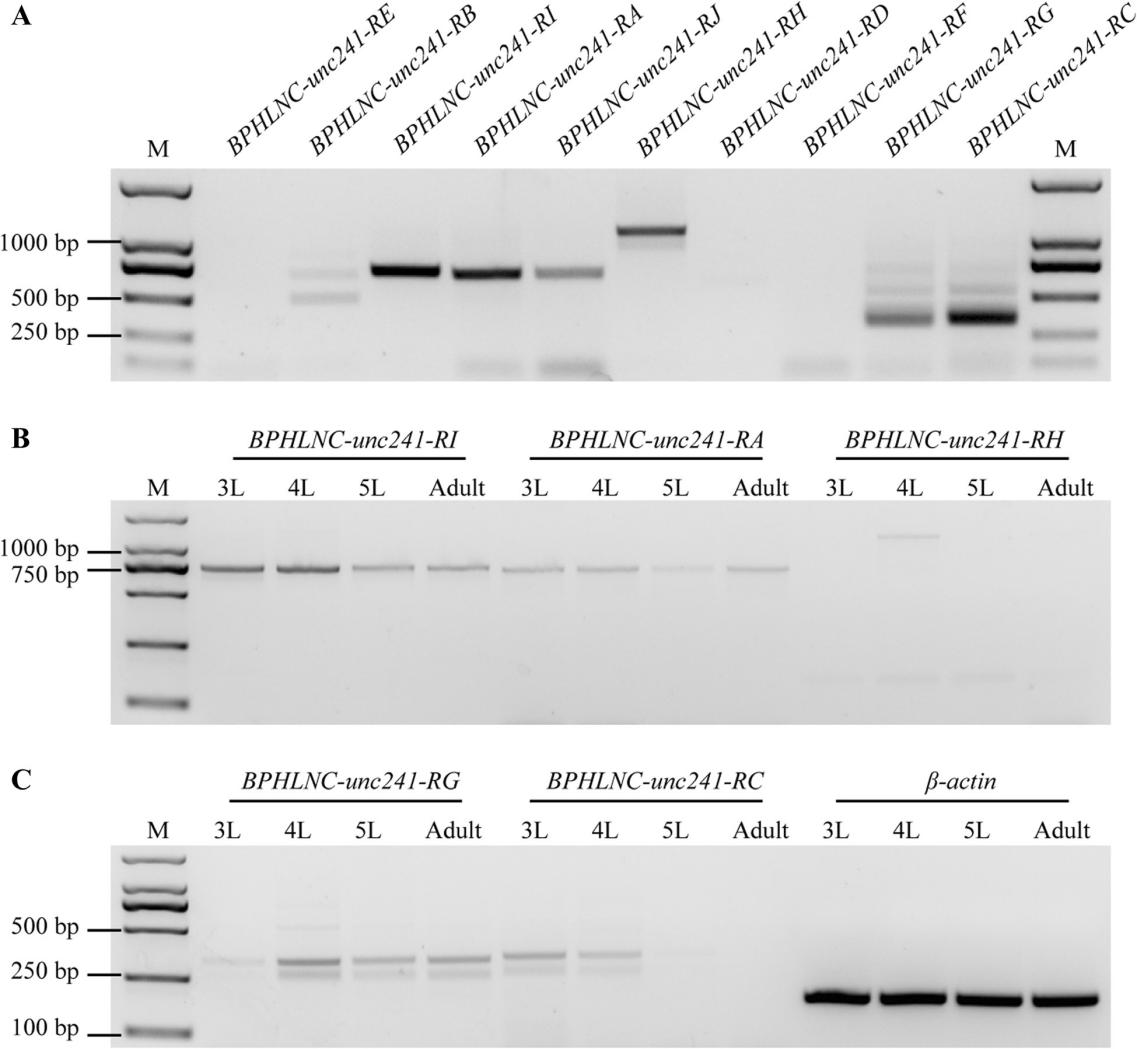
RT-PCR validation of alternatively spliced transcripts of *BPHLNC-unc241*. Isoform-specific primers were designed for ten isoforms. Five of them were successfully amplified followed by sequencing. The PCR product size and the sequencing result of variant J was not as expect (**a**). The expressions of five isoforms in the third instar to fifth instar nymph and adult were measured, suggesting transcript variants vary in their expression profiles (**b**,**c**)

Only 19.9 % of *N. lugens* lncRNA genes had alternative splicing (AS) (Additional file 4: Figure S3), suggesting that AS is not prevalent in lncRNA genes. However, there were some exceptions. Some lncRNAs showed abundant alternative splicing events. *BPHOGS10002343-OT* and *BPHOGS10000007-OT* had 11 isoforms, and *BPHLNC-unc241* had ten isoforms (Fig. 4). We selected *BPHLNC-unc241* to validate alternatively spliced transcripts. Isoform-specific primers of ten alternatively spliced transcripts were designed. Out of them, five isoforms were successfully amplified and were confirmed by sequencing (Fig. 5a). Though there was a band in the lane of variant J, the PCR product size was not correct and the sequencing result was not as expected. Possibly because of spatiotemporal expression of alternatively spliced isoforms, other four isoforms were not detected. We selected the 3rd, 4th, 5th instar nymph and adult to study the expression of five isoforms. Semi-quantitative PCR indicated that *BPHLNC-unc241-RA* and *BPHLNC-unc241-RI* were highly expressed in the 3rd and the 4th instar nymph but lowly in other stages. *BPHLNC-unc241-RC* was expressed only in the 3rd and 4th instar whereas *BPHLNC-unc241-RH* only in the 4th instar. The mRNA abundance of *BPHLNC-unc241-RG* was high in all samples except the 3rd instar nymph (Fig. 5b). These results suggest that alternatively spliced isoforms of *BPHLNC-unc241-R* have different expression profiles and might have differential functions during development.

### Specifically-expressed and differentially-expressed lncRNAs

**Fig. 6:**
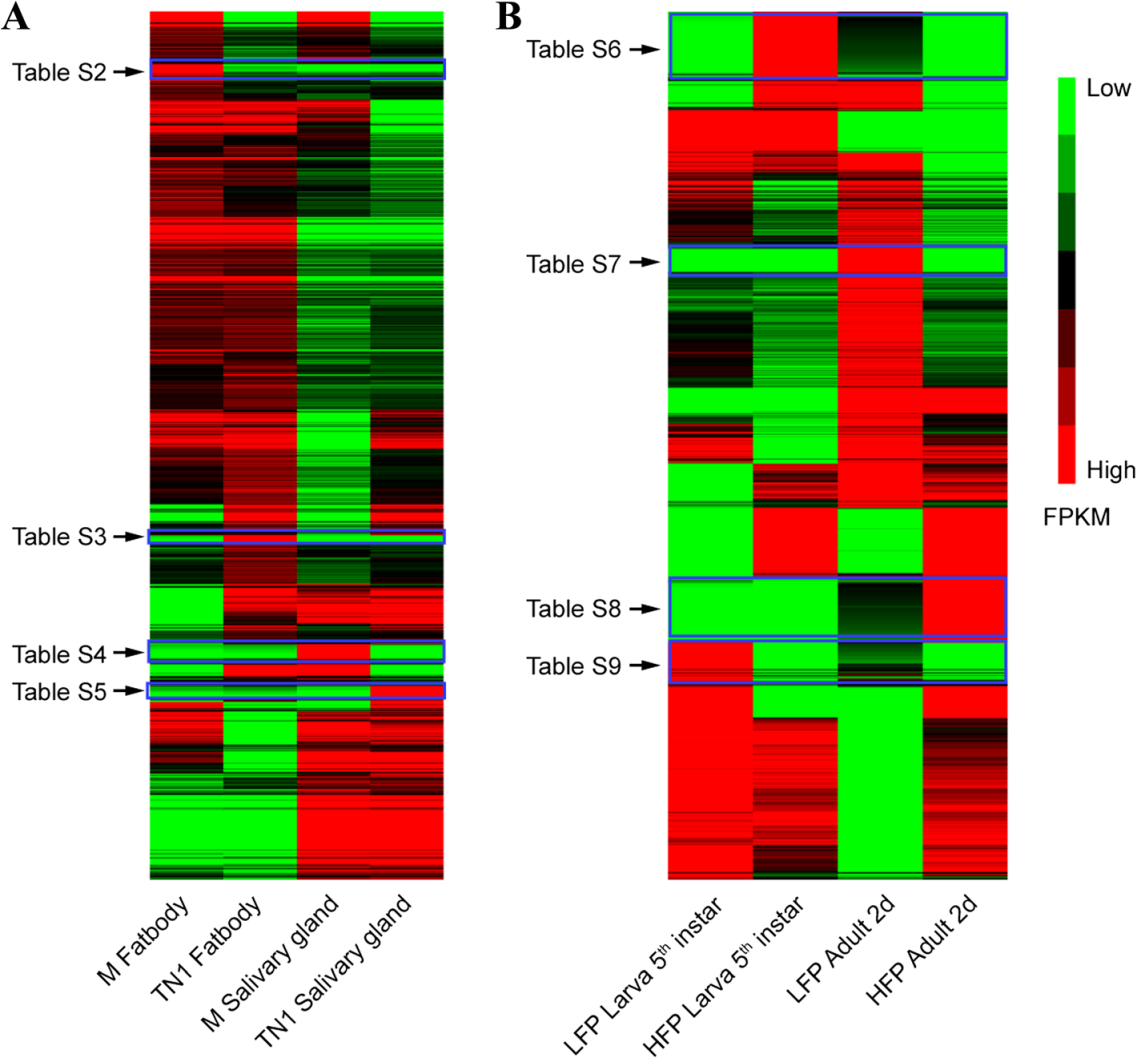
The heatmap of the lncRNA expression patterns in virulent and fecund populations. **a** Expression profile changes of lncRNA transcripts across the fat body and salivary gland in the Mudgo and TN1 populations. **b** Hierarchical clustering of expressional abundance of lncRNA transcripts in the nymph and adult of the HFP and LFP population. The lncRNAs showing tissue specific expression in the Mudgo and TN1 populations are listed in Additional file 6, 7, 8 and 9: Tables S2, S3, S4 and S5. The lncRNAs specifically expressed in the fifth instar nymph and adult were listed in Additional file 10, 11, 12 and 13: Tables S6, S7, S8 and S9

We estimated the transcript abundance of lncRNAs using raw reads of all 12 *N. lugens* RNA-Seq datasets. The results indicated that most lncRNAs were expressed in almost all *N. lugens* transcriptomes (Fig. 6). Interestingly, we found that the number of specifically expressed lncRNAs was higher than that of differentially expressed lncRNAs (Additional file 5: Table S1). In total, 19–148 lncRNAs were specifically-expressed whereas 0–10 lncRNAs were differentially-expressed in LFP, HFP, TN1, Mudgo, I87i and C89i population (Additional files 6, 7, 8, 9, 10, 11, 12 and 13: Table S2, S3, S4, S5, S6, S7, S8 and S9). There were 146 specifically-expressed lncRNAs and ten differentially highly-expressed lncRNAs in the adult of HFP population. One hundred and forty-eight specifically-expressed and two highly-expressed lncRNAs were found in the fifth instar nymph of HFP population. In the adult and the fifth instar larvae of the LFP population, 58 and 76 lncRNAs were specifically-expressed while three and one lncRNAs were highly-expressed. In the fat body and salivary gland of the Mudgo and TN1 populations, there were only 21–42 specifically-expressed lncRNAs and 0–4 differentially-expressed lncRNAs. The high numbers of specifically-expressed lncRNAs in LFP and HFP populations suggested that lncRNAs might play key roles in the fecundity of *N. lugens*.

### LncRNAs associated with fecundity

**Fig. 7:**
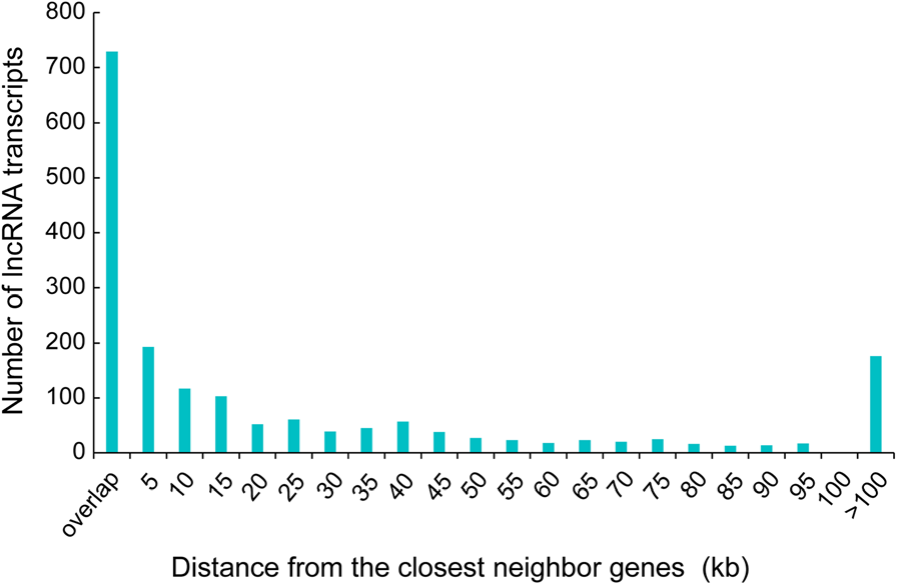
The genome location of lncRNA in *N. lugens*, demonstrating that lncRNAs tend to located adjacently to protein-coding genes (<5 Kb). A high number of lncRNAs overlapped with protein-coding genes

**Fig. 8:**
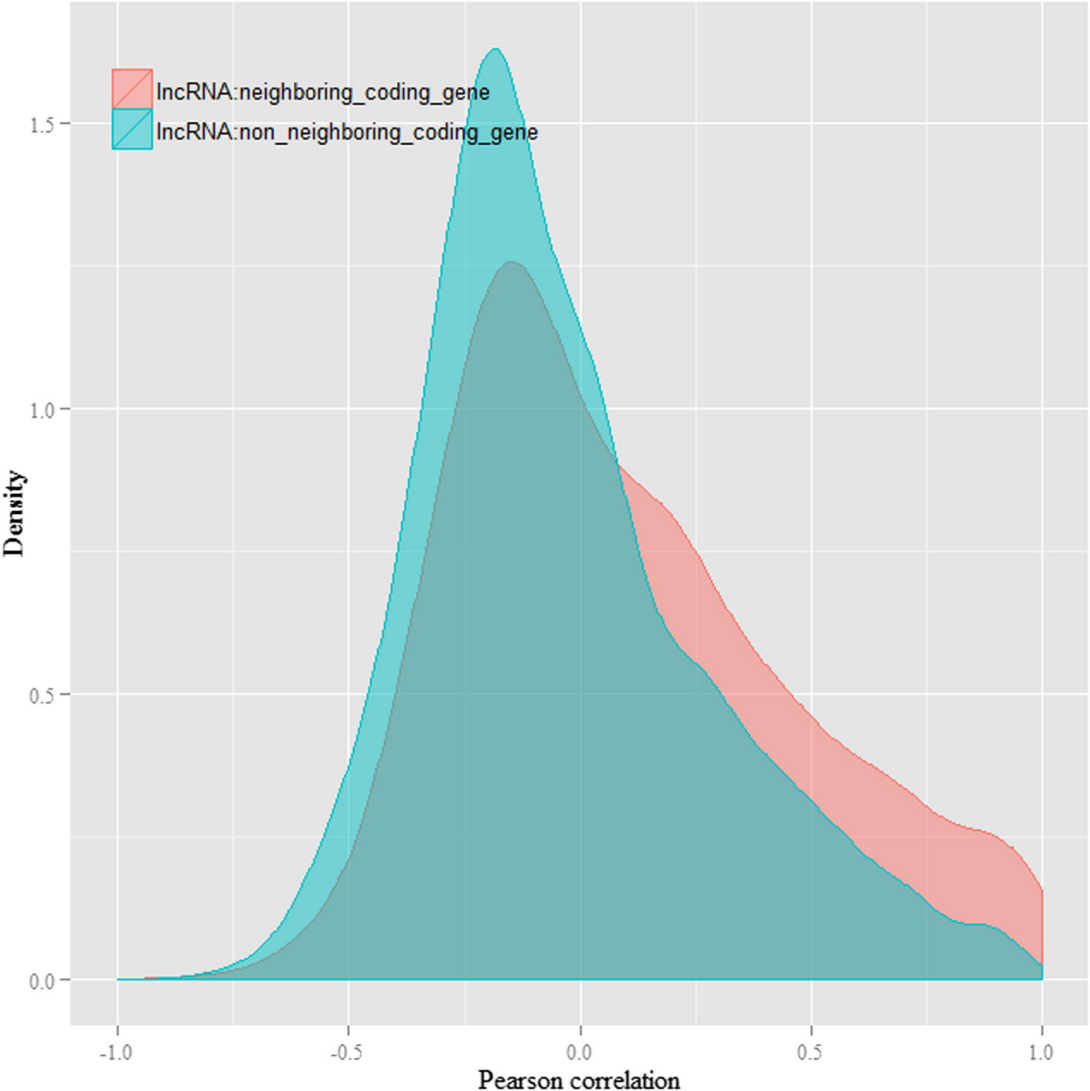
The distribution of Pearson product–moment correlation coefficient between lncRNA and protein-coding genes. lncRNAs had higher coefficients with their neighboring protein-coding genes than with non-neighboring genes, suggesting that lncRNAs tends to be co-expressed with neighboring genes. The density in Y-axis means the area under the curve of a density function represents the probability of getting an x value between a range of x values

**Fig. 9:**
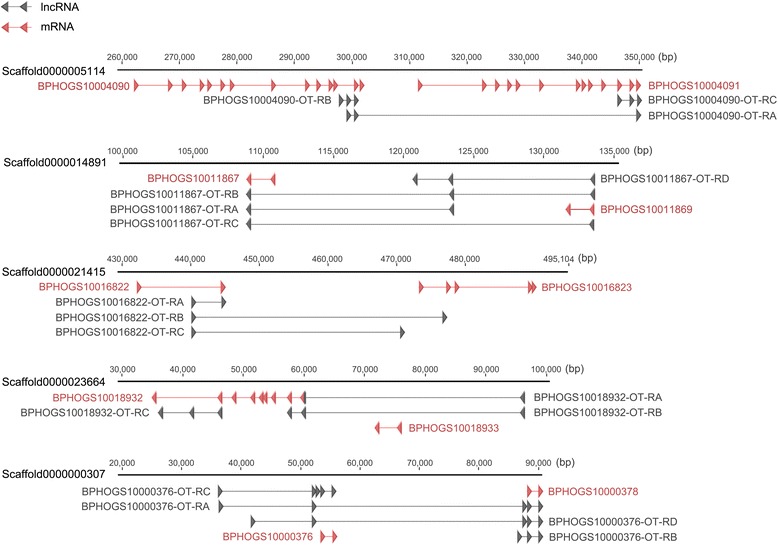
Gene structures of five lncRNA genes. These lncRNA genes had multiple isoforms and overlapped with two adjacent protein-coding genes

It has been reported that lncRNAs tend to be co-expressed with the overlapping genes or adjacent genes by sharing a same primary transcript. Some lncRNAs interact with overlapping or adjacent genes by chromosome modeling [32]. We found that 30 % of *N. lugens* lncRNA genes overlapped with known protein-coding genes (Fig. 7). In different samples, there were a number of lncRNAs located within < 5 Kb of protein-coding genes (Table 2). The Pearson correlation r of transcripts abundance between lncRNAs and their adjacent protein-coding genes was 0.1, which was significantly higher than the Pearson correlation r (−0.03) between lncRNAs and randomly selected coding genes (*P* < 0.01, *T*-test, Fig. 8), suggesting that lncRNAs tend to be co-expressed with their adjacent genes. Interestingly, five lncRNA genes overlapped with two protein-coding genes (Fig. 9).

**Table 2:** Specifically expressed *N. lugens* lncRNA in varied RNA-seq datasets

Samples	Specific expressed lncRNA distribution according to the distance with the closest gene (lncRNA transcript number/closest gene number)			
	overlap	<5 k	<10 k	>10 k
HFP adult 2 d	36/33	10/9	9/8	41/54
LFP adult 2 d	17/17	3/4	4/4	9/9
HFP 5^th^ instar larva	37/36	20/22	8/9	44/56
LFP 5^th^ instar larva	19/18	11/11	6/6	22/25
Mudgo fat body	6/6	5/5	1/1	7/7
TN1 fat body	8/8	3/3	1/1	3/5
Mudgo salivary gland	7/7	4/5	5/5	11/18
TN1 salivary gland	8/8	2/2	1/1	7/9

In the HFP population, there were 6992 protein-coding genes that were co-expressed with lncRNAs, among which 46 protein-coding genes involve in energy metabolism. In the LFP population, there were 7089 protein-coding genes that were co-expressed with lncRNAs, among which 48 protein-coding genes involve in energy metabolism. The protein coding genes associated with energy metabolisms were not located adjacently or overlapped with any lncRNAs.

**Fig. 10:**
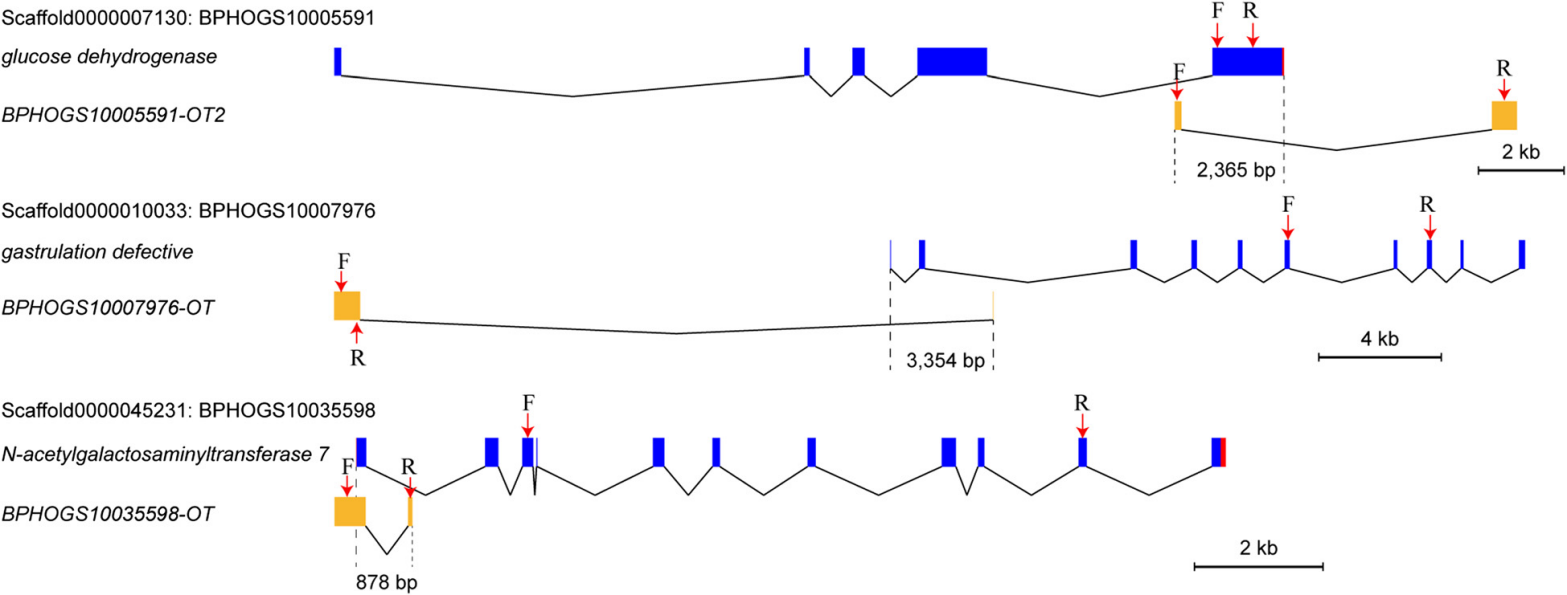
Exon and intron structures of three lncRNA genes that were specifically expressed in the HFP or LFP population. These lncRNAs overlapped with reproduction-associated protein genes encoding glucose dehydrogenase, gastrulation defective, and *GALNT7*. F: Forward primer, R: Reverse primer

We found that three lncRNA genes overlapped with reproduction-associated genes (Fig. 10). Two lncRNAs (*BPHOGS10035598-OT* and *BPHOGS100007976-OT*) were specifically-expressed in the fifth instar nymph of the HFP population. One lncRNA (*BPHOGS10005591-OT2*) was specifically-expressed in the fifth instar nymph of the LFP population. *BPHOGS10005591-OT2* overlapped with the glucose dehydrogenase (*GLD*) gene at the 3′ region comprising 2365 bp. *GLD* is essential for sperm storage in adult female of *D. melanogaster. BPHOGS100007976-OT* was located at the 5′-upstream of the gastrulation defective gene and overlapped with this gene for 3354 bp. The gastrulation defective gene encodes a serine protease that cleaves and activates protein SNAKE. The activated SNAKE cleaves and activates protein EASTER. This series of activations controls the embryo dorsoventral polarity. *BPHOGS10035598-OT* overlapped with the N-acetylgalactosaminytransferase 7 gene (*GALNT7*) at its 5′-end for 878 bp. *GALNT7* participates in reproductive regulation in *D. melanogaster*.

**Fig. 11:**
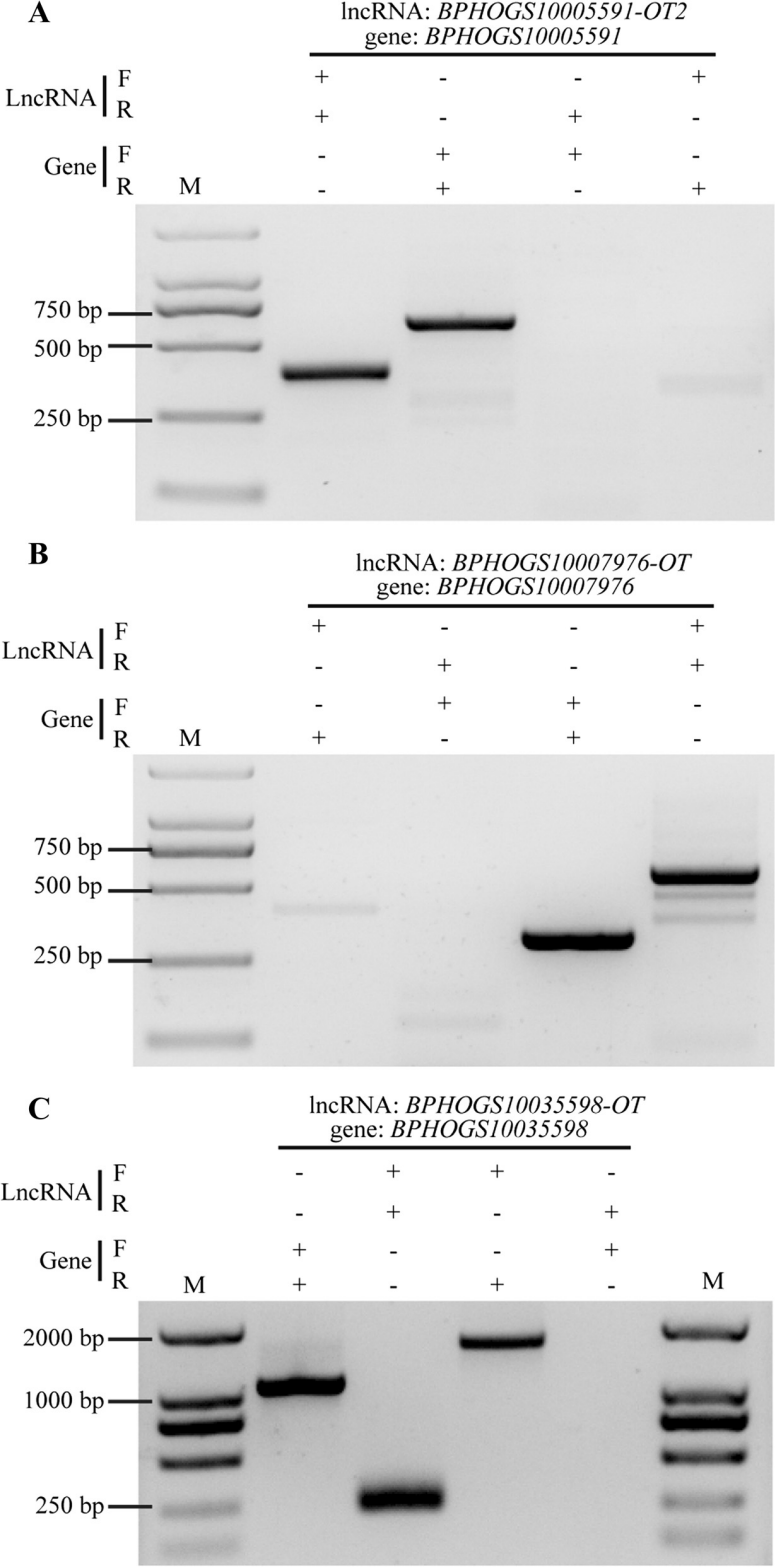
Amplification of three lncRNAs overlapped with reproduction-associated protein genes. Different combinations of primers pairs indicated that *BPHOGS10005591-OT2* (**a**) and *BPHOGS10007976-OT* (**b**) were independently transcribed whereas *BPHOGS10035598-OT* (**c**) might share a same transcript with its adjacent protein-coding gene *BPHOGS10035598*

We carried out RT-PCR to confirm the transcription of three lncRNAs and their adjacent protein-coding genes using a wild population. The different combinations of primer-pairs were used to examine the transcripts. The results suggested that the identified OT-type of lncRNAs were not the artifacts of full-length coding sequences. *BPHOGS10005591-OT2* and *BPHOGS10007976-OT* were transcribed independently from there adjacent protein-coding genes whereas *BPHOGS10035598-OT* might share a same transcript with its adjacent protein-coding gene *BPHOGS10035598* (Fig. 11).

## Discussion

We identified 2439 lncRNA transcripts corresponding to 1882 loci from 12 *N. lugens* RNA-seq datasets including four transcriptome datasets of LFP or HFP fecundity strains, five transcriptomes from the fat body, salivary gland, and antennae of the virulence strain and three other different populations. BLASTN searching of *N. lugens* lncRNAs against the NCBI nr and NONCODE databases found no highly similar sequences, demonstrating that lncRNAs lack sequence conservation. However, it has been reported that an lncRNA, *yar*, is conserved in *Drosophila* species [28]. The lncRNA secondary structures of *A. gambiae* were conserved within the Gambiae complex [19]. Here, we found that structural features of *N. lugens* lncRNAs are similar to mammalian counterparts. We also performed RT-PCR and strand-specific PCR to confirm the expression of 20 randomly selected lncRNAs. The results indicated that >80 % identified lncRNAs were reliable. They were unlikely to be the artifacts of full-length coding sequences.

It has been reported that the functions of lncRNAs can be inferred by analyzing their co-expression networks and genome locations [32, 33]. In *D. melanogaster*, the lncRNA CRG positively regulates its neighboring Ca(2+)/calmodulin-dependent protein kinase, which is essential for locomotor activity and climbing ability [29]. 19–148 *N. lugens* lncRNAs were specifically-expressed in the HFP, LFP, TN1 and Mudgo populations, respectively. However, less than ten lncRNAs were differentially-expressed in various samples. At least three specifically-expressed lncRNAs, *BPHOGS10005591-OT2*, *BPHOGS100007976-OT*, and *BPHOGS10035598-OT,* overlap with reproduction-associated genes that have important functions in sperm storage and embryo dorsoventral polarity. These lncRNAs are also co-expressed with the reproduction-associated genes. According to the evidence of co-expression and genome-location, lncRNAs might have important roles in regulating fecundity in *N. lugens*. We did not find any lncRNA to be located adjacently to protein-coding genes associated with the virulence adaptation of *N. lugens*, possibly because the mechanism of virulence remains largely unknown. High fecundity and virulence adaptability are two main factors that underlie the great damage caused by *N. lugens* [31, 34]. We found indication that lncRNA might participate in the regulation of at least one of these two important biological processes, which should provide new insights into developing alternative eco-friendly pest-control policies for the rice brown planthopper. However, it should be noticed that the evidence presented here are not direct.

## Conclusions

A computational pipeline was constructed to identify lncRNA genes from the rice brown planthopper, yielding 2439 lncRNA transcripts corresponding to 1882 loci. Insect lncRNAs shared similar structural gene features with mammalian lncRNAs. 19–148 lncRNAs were specifically-expressed in high fecundity or low fecundity populations. At least three of them were overlapped with reproductive-associated genes. In terms of genome-location and gene-expression, we presented some indications that lncRNAs might play important roles in fecundity and virulence adaptation in *N. lugens*. Function analysis of lncRNAs is required to elucidate their roles in regulating fecundity and virulence adaptation.

## Methods

### Insects

The rice brown planthoppers were collected from rice fields in Nanjing area, Jiangsu Province, China and maintained on rice seedlings at 27 ± 1 °C, under a 16-h light/8-h dark photoperiod and 70–80 % relative humidity. The insects were transferred to fresh seedlings every 5–7 days to ensure sufficient nutrition.

### Data

The draft genome sequences of *N. lugens* were kindly provided by Professor Chuanxi Zhang in Zhejiang University [35]. We annotated the genome sequences using the OMIGA pipeline [36] and deposited the annotation information in InsectBase (http://www.insect-genome.com/). We obtained 12 transcriptomes of *N. lugens*, including transcriptome of the 5th instar nymph of a low fecundity population (LFP) and a high fecundity population (HFP), two-days old adults of LFP and HFP population and a wild population. These populations had similar genetic background because they were selected from a starting population. All insects were maintained at same conditions and the transcriptomes were sequenced with a same protocol. The detailed method procedures of sequencing the transcriptome of LFP, HFP and the control population have been reported in [37]. The other seven transcriptomes included the salivary glands of the Mudgo and TN1 population, the fat body of the Mudgo and TN1 population, the antennal of the TN1 population, I87i and C89i population. The transcriptome data were downloaded from the NCBI SRA database [38, 39]. The accession numbers were SRX276866 (the salivary glands of the Mudgo population), SRX276865 (the salivary glands of the TN1 population), SRX360414 (the fat body of the Mudgo population), SRX360412 (the fat body of the TN1 population), SRX290503 (the antennal of the TN1 population), DRX014540 (I87i strain), and DRX014541 (C89i strain).

All these transcriptomes were sequenced using the Illumina sequencing platform (GAII). Random hexamers were used in the cDNA synthesis. Total RNA were used for sequencing the transcriptomes of TN1, Mudgo, I87i and C89i populations whereas poly(A) + RNA were used for constructing the cDNA libraries in sequencing the transcriptomes of LFP, HFP and the control population. In this case, only those lncRNAs with poly (A) tails can be found from the transcriptomes of LFP, HFP and the control populations. It should be noted that many lncRNAs do not have poly (A) tail. These lncRNAs cannot be found from these transcriptomes.

### Developing a computational pipeline to identify lncRNAs

A computational pipeline was constructed to identify lncRNA genes from the RNA-seq data. First, the RNA-seq reads of 12 *N. lugens* RNA-seq datasets were mapped to the genome using TopHat [40]. For the first run, the reads from each RNA-seq dataset were mapped to the genome independently. The junction outputs from each RNA-seq dataset were pooled together as a Pooled Junction Set. This allowed TopHat to use junction information from all RNA-seq datasets. For the second run, TopHat was run on each RNA-seq dataset separately using the Pooled Junction Set. The output of this second run produced the final junction set for transcript assembly using Cufflinks [3]. Second, the assembled transcripts of the 12 RNA-seq datasets were combined together by Cuffcompare, using *N. lugens* genome-annotation information. The transcripts that satisfied two criteria were retained: length ≥ 200 nt and exon numbers ≥ 2. This step produced 94,388 transcripts corresponding to 43,474 loci. Third, their protein coding potentials were examined by the software getorf (http://emboss.sourceforge.net/apps/cvs/emboss/apps/getorf.html). Transcripts with an open reading frame ≥ 300 nt were removed. Fourth, the remaining transcripts were searched against the SWISS-PROT database using BLASTX. Those transcripts that had BLAST hits with known proteins (*e*-value < 0.001) were regarded as mRNA transcripts and removed. We also removed the putative untranslated region fragments of known mRNA transcripts by sequence alignments, producing 9392 transcripts corresponding to 6734 loci. Fifth, all 9392 transcripts were estimated by the software Coding Potential Calculator (CPC, http://cpc.cbi.pku.edu.cn/). Only those transcripts with a CPC score ≤ −1 were kept, yielding 6175 transcripts corresponding to 4490 loci. Sixth, the remaining transcripts were used to search against the Pfam database using the software Hmmer [41]. Those transcripts that had the potential to encode conserved domains or motifs were removed. In the last step, we removed known tRNAs), ribosomal RNAs (rRNAs), snoRNA, and small nuclear RNAs (snRNAs) by searching the Rfam database using Infernal [42] and BLASTN against the NONCODE database [43], producing the final lncRNA gene sets.

### LncRNA gene expression analysis in 12 *N. lugens* RNA-seq datasets

The transcript abundance of the identified lncRNA genes were estimated by counting reads and normalizing with the software Cuffdiff [3], which used *T*-test to measure the significance of the expressional difference. A heatmap was produced by analyzing the expression abundance of lncRNA genes with the software Clustering [44]. The average linkage method was used and the results were viewed using Java TreeView [45]. If the expression of a lncRNA meets following criteria, we defined it as the specifically-expressed lncRNA: 1) the expression is > 3 FPKM in one sample whereas it is < 1 FPKM in other samples; 2) the FPKM of this lncRNA in one sample is at least 10-fold higher than those in other samples. For finding differentially-expressed lncRNAs, the cutoff was set as *p*-value <0.01 and *q*-value < 0.05. *q*-value means the FDR-adjusted *p*-value of the test statistic.

### Co-expression analysis of protein-coding genes and lncRNAs

Co-expression analysis was performed between lncRNAs and protein-coding genes using all 12 transcriptome RNA-seq datasets. Pearson product–moment correlation coefficient was used to estimate the co-expression relationship by using a R script. The lncRNA: mRNA relationship with |r| > 0.8 were treated as the strong correlation.

### Structural gene features of *N. lugens* lncRNAs

Gene structures of lncRNA genes were constructed by aligning lncRNAs with the *N. lugens* genome. The protein-coding gene information was obtained by the OMIGA annotation. The lengths of exons and introns were calculated. We wrote a Perl scalable vector graphics module to draw the exon-intron structures of the lncRNA genes. The software Geneious was used to show the transcript structure of lncRNA and protein-coding genes [46].

### Total RNA isolation and cDNA synthesis

The third to the fifth instar of *N. lugens* nymph and adult were chose for gene expression analysis. Total RNA was extracted from 50 individuals of a wild population using the TRIzol® reagent, following the manufacturer’s instructions (Life Technologies, CA, USA). RNA integrity was checked by electrophoresis using 1.2 % agarose gels. The RNA purity was examined using a Nanodrop spectrophotometer (Thermo Fisher Scientific, Waltham, MA, USA). The cDNA synthesis was performed following the manufacturer’s instructions of the PrimeScript™ RT reagent Kit with gDNA Eraser (Takara, Kyoto, Japan). Random primers were used in the cDNA synthesis for RT-PCR amplification of lncRNAs. Gene-specific primers (GSP) were used in the cDNA synthesis for the strand-specific RT-PCR.

### RT-PCR

We randomly selected 20 lncRNA genes for validation. The rice brown planthoppers from a wild population were used for extracting total RNA. The strand-specific RT-PCR was used to determine the transcript orientation. In the cDNA synthesis, three reactions were used: Forward (F) primer with reverse transcriptase (RT), reverse (R) primer with RT, both F and R primers without RT. To validate the alternative splicing of lncRNAs, we selected *BPHLNC-unc241* for isoform-specific PCR. This lncRNA gene has ten alternatively spliced transcripts. The transcription of three reproduction-associated protein-coding genes (BPHOGS10005591*, BPHOGS10007976* and *BPHOGS10035598*) and their overlapping lncRNA genes (*BPHOGS10005591-OT2*, *BPHOGS10007976-OT* and *BPHOGS10035598-OT*) were also confirmed by RT-PCR.

The primers were designed using an Integrated DNA Technologies online tool (IDT, Coralville, IA, USA; http://www.idtdna.com/Scitools/) and the primer sequences are shown in Additional files 14, 15 and 16: Tables S10, S11 and S12. Premix Taq® Version 2.0 kit (Takara) was used for the PCR reactions, which were performed in a T100 thermal cycler (Bio-Rad, Hercules, CA, USA). PCR conditions were 94 °C for 5 min; followed by five cycles of 94 °C for 30 s, 60 °C (reduced by 1 °C/cycle) for 30 s and 72 °C for 1 min; and then 28 cycles of 94 °C for 30 s, 50 °C for 30 s, and 72 °C for 1 min. The last step was followed by final extension at 72 °C for 10 min. The PCR products were checked by electrophoresis using 1.5 % agarose gels. The PCR products were purified by using Wizard HSV Gel (Promega, Madison, WI, USA), following the manufacturer’s instructions. The PCR products were sequenced by the GeneScript Company (Nanjing, China).

## Additional files

Additional file 1:
**The sequences of identified lncRNAs in FASTA format.** (FASTA 2166 kb) Additional file 2: Figure S1. The relationship between the numbers of identified lncRNA genes and read counts in varied samples of *N. lugens*. The high coverage of RNA-seq was positively related with the numbers of detected lncRNAs. However, in the adult of HFP population, the coverage was low but a high number of lncRNAs were found. LFP: low fecundity population, HFP: high fecundity population. TN1: avirulent Taichung Native 1 host strain, Mudgo: virulent (carrying the resistance gene bph1) host strain, I87i: Izumo87 strain, C89i: Chikugo89 strain. (TIFF 133 kb) Additional file 3: Figure S2. RT-PCR validation of 20 randomly selected lncRNAs. Seventeen lncRNAs were successfully amplified and confirmed by sequencing. The PCR product in lane 15, *BPHLNC-unc536*, was not correctly amplified. Two lncRNAs, *BPHLNC-unc525* and *BPHLINC406,* were not amplified and were not shown in the figure. Lane 1–18: *BPHOGS10028742-AS-RA*, *BPHLINC074-RA*, *BPHOGS10028378-AS-RA*, *BPHOGS10006054-OT-RA*, *BPHLINC250-RA*, *BPHOGS10022296-OT-RA*, *BPHLNC-unc280-RA*, *BPHLINC164-RA, BPHOGS10026274-IT-RA, BPHOGS10006052-AS-RA*, *BPHOGS10017161-OT-RA*, *BPHOGS10027736-OT-RB*, *BPHOGS10003291-OT-RA*, *BPHLNC-unc005-RA*, *BPHLNC-unc536-RA*, *BPHOGS10000919-OT-RA*, *BPHOGS10035448-AS-RA*, *BPHOGS10030139-OT-RA*, respectively. (TIFF 4040 kb) Additional file 4: Figure S3. Alternative splicing of identified lncRNAs in *N. lugens*. Most lncRNAs had only one isoforms. Only 19.9 % lncRNAs had multiple isoforms. (TIFF 63 kb) Additional file 5: Table S1. The number of specifically-expressed and differentially-expressed lncRNAs in various populations (DOCX 14 kb) Additional file 6: Table S2. Specifically expressed lncRNAs in the fat body of Mudgo strain of *N. lugens*. (XLSX 9 kb) Additional file 7: Table S3. Specifically expressed lncRNAs in the fat body of TN1 strain of *N. lugens*. (XLSX 9 kb) Additional file 8: Table S4. Specifically expressed lncRNAs in the salivary gland of Mudgo strain of *N. lugens*. (XLSX 10 kb) Additional file 9: Table S5. Specifically expressed lncRNAs in the salivary gland of TN1 strain of *N. lugens*. (XLSX 10 kb) Additional file 10: Table S6. Specifically expressed lncRNAs in the 5th instar nymph of HFP population (XLSX 16 kb) Additional file 11: Table S7. Specifically expressed lncRNAs in the adult of LFP population. (XLSX 12 kb) Additional file 12: Table S8. Specifically expressed lncRNAs in the adult of HFP population. (XLSX 15 kb) Additional file 13: Table S9. Specifically expressed lncRNAs in LFP the 5th instar nymph of LFP population (XLSX 13 kb) Additional file 14: Table S10. Primers used for RT-PCR and strand-specific PCR. Both two pairs of primers were used for RT-PCR validation. One pair of primers was used for strand-specific PCR for determining transcript orientations. *: the primer used for strand specific PCRs. (DOCX 24 kb) Additional file 15: Table S11. Primers used for RT-PCR validation of alternatively spliced isoforms of *BPHLNC-unc241*. (DOCX 13 kb) Additional file 16: Table S12. Primers used for RT-PCR validation of three lncRNA genes and their overlapping protein coding genes. (DOCX 12 kb)
