# Supplementary figures and images for: Genome-wide identification of long noncoding RNA genes and their potential association with fecundity and virulence in rice brown planthopper, Nilaparvata lugens
Source: BMC Genomics. 2015 Oct 5;16:749. doi: 10.1186/s12864-015-1953-y (PMC4594746; doi:10.1186/s12864-015-1953-y)

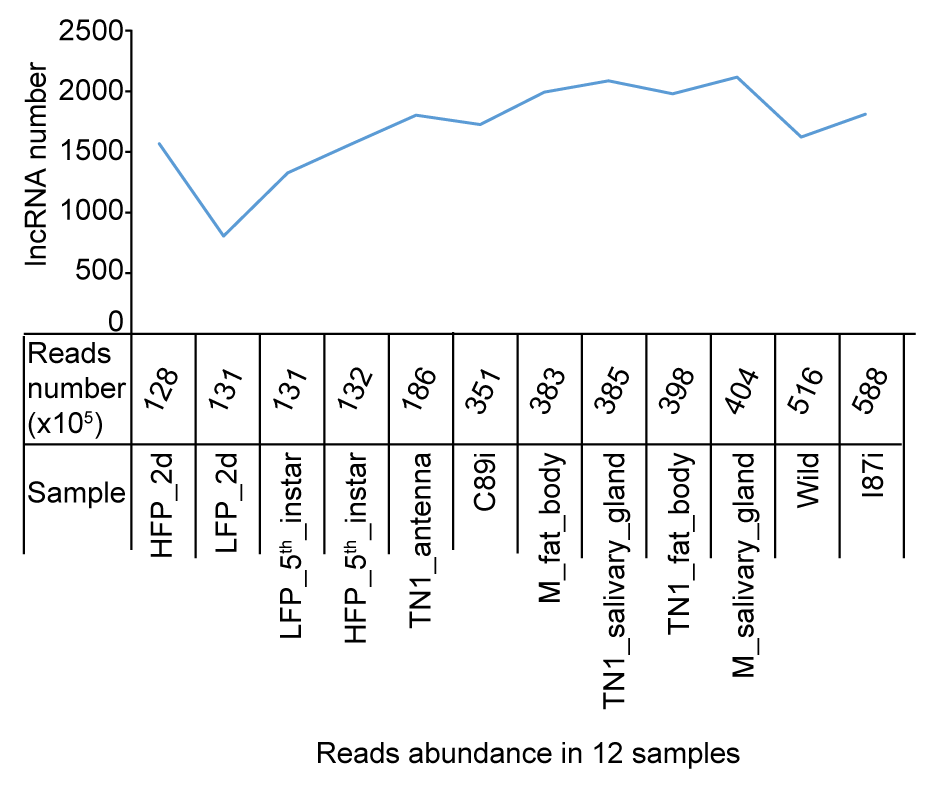

Supplement: Additional file 2: Figure S1. — The relationship between the numbers of identified lncRNA genes and read counts in varied samples of N. lugens. The high coverage of RNA-seq was positively related with the numbers of detected lncRNAs. However, in the adult of HFP population, the coverage was low but a high number of lncRNAs were found. LFP: low fecundity population, HFP: high fecundity population. TN1: avirulent Taichung Native 1 host strain, Mudgo: virulent (carrying the resistance gene bph1) host strain, I87i: Izumo87 strain, C89i: Chikugo89 strain. (TIFF 133 kb) [file 12864_2015_1953_MOESM2_ESM.tif]

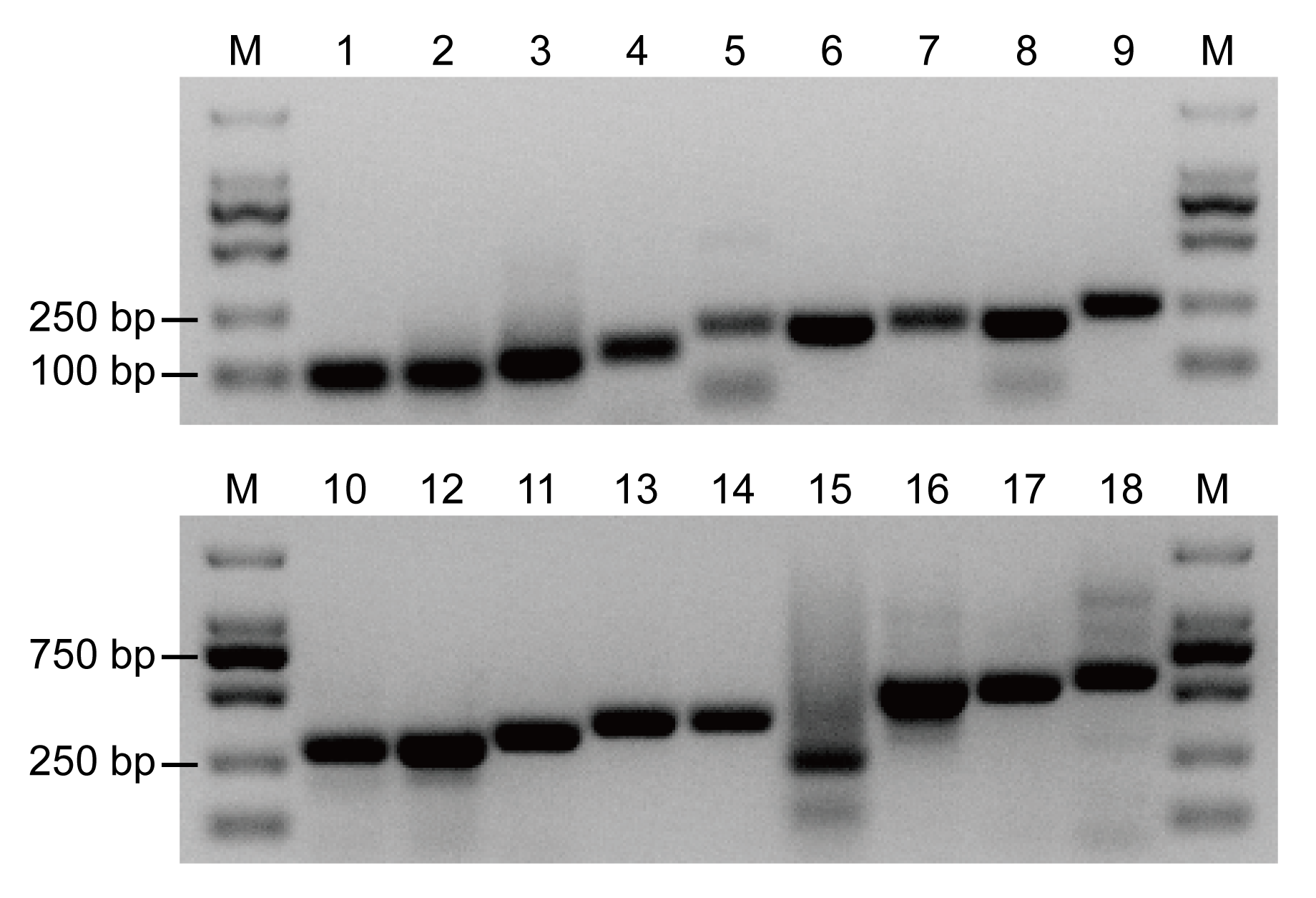

Supplement: Additional file 3: Figure S2. — RT-PCR validation of 20 randomly selected lncRNAs. Seventeen lncRNAs were successfully amplified and confirmed by sequencing. The PCR product in lane 15, BPHLNC-unc536, was not correctly amplified. Two lncRNAs, BPHLNC-unc525 and BPHLINC406, were not amplified and were not shown in the figure. Lane 1–18: BPHOGS10028742-AS-RA, BPHLINC074-RA, BPHOGS10028378-AS-RA, BPHOGS10006054-OT-RA, BPHLINC250-RA, BPHOGS10022296-OT-RA, BPHLNC-unc280-RA, BPHLINC164-RA, BPHOGS10026274-IT-RA, BPHOGS10006052-AS-RA, BPHOGS10017161-OT-RA, BPHOGS10027736-OT-RB, BPHOGS10003291-OT-RA, BPHLNC-unc005-RA, BPHLNC-unc536-RA, BPHOGS10000919-OT-RA, BPHOGS10035448-AS-RA, BPHOGS10030139-OT-RA, respectively. (TIFF 4040 kb) [file 12864_2015_1953_MOESM3_ESM.tif]

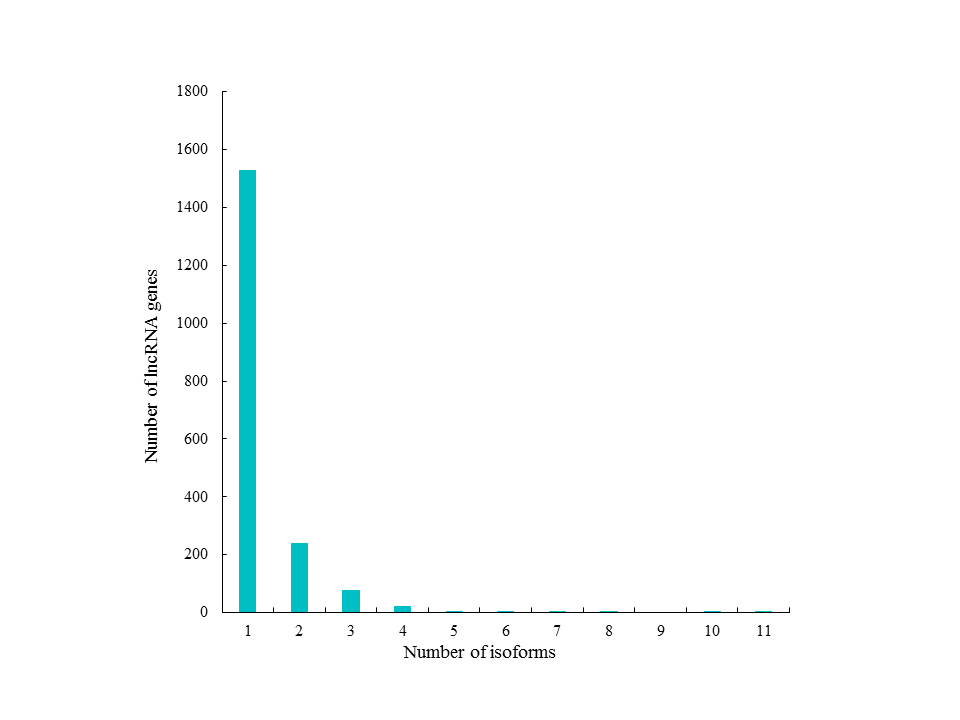

Supplement: Additional file 4: Figure S3. — Alternative splicing of identified lncRNAs in N. lugens. Most lncRNAs had only one isoforms. Only 19.9 % lncRNAs had multiple isoforms. (TIFF 63 kb) [file 12864_2015_1953_MOESM4_ESM.tif]
