# Supplementary material for: Genome-wide identification of long noncoding RNA genes and their potential association with fecundity and virulence in rice brown planthopper, Nilaparvata lugens
Source: BMC Genomics. 2015 Oct 5;16:749. doi: 10.1186/s12864-015-1953-y (PMC4594746; doi:10.1186/s12864-015-1953-y)
Supplement: Additional file 5: Table S1. — The number of specifically-expressed and differentially-expressed lncRNAs in various populations (DOCX 14 kb) [file 12864_2015_1953_MOESM5_ESM.docx]

**Table S12. The number of specifically-expressed and highly-expressed lncRNAs in various populations.**

| Populations | | Development stages/tissues | Specifically-expressed | Differentially expressed |
| --- | --- | --- | --- | --- |
| HFP | Adult | | 146 | 10 |
|  | Nymph | | 148 | 2 |
| LFP | Adult | | 58 | 3 |
|  | Nymph | | 76 | 1 |
| Mudgo | Salivary gland | | 41 | 2 |
|  | Fatbody | | 24 | 0 |
| TN1 | Salivary gland | | 27 | 4 |
|  | Fatbody | | 19 | 0 |
