# Supplementary material for: Genome-wide identification of long noncoding RNA genes and their potential association with fecundity and virulence in rice brown planthopper, Nilaparvata lugens
Source: BMC Genomics. 2015 Oct 5;16:749. doi: 10.1186/s12864-015-1953-y (PMC4594746; doi:10.1186/s12864-015-1953-y)
Supplement: Additional file 14: Table S10. — Primers used for RT-PCR and strand-specific PCR. Both two pairs of primers were used for RT-PCR validation. One pair of primers was used for strand-specific PCR for determining transcript orientations. *: the primer used for strand specific PCRs. (DOCX 24 kb) [file 12864_2015_1953_MOESM14_ESM.docx]

**Table S10 Primers used for RT-PCR and strand-specific PCR. Both two pairs of primers were used for RT-PCR validation. One pair of primers was used for strand-specific PCR for determining transcript orientations.**

| lncRNA genes | lncRNA length (bp) | Forward primers (5’-3’) | Reverse primers (5’-3’) | product size (bp) | Transcript Orientation |
| --- | --- | --- | --- | --- | --- |
| *BPHOGS10028742-AS-RA* | 322 | CTCTCGGTGTGGCATTTCTAA | GGAAGAAGAATCGTTCGGTCA | 104 | Antisense* |
|  |  | CAAGTGTGGACCGTATGAAGAG | TCGGTCACCACAAATCGTATC | 166 |  |
| *BPHLINC074-RA* | 212 | CTCATGTTGGTGAAGGAGTAAGA | CAGGAACATTATTGAAGCCTGAAG | 108 | Antisense* |
|  |  | CTTCCAACGAATACACTGCTTTG | TGAAGCGCCTCCATGATTT | 122 |  |
| *BPHOGS10028378-AS-RA* | 310 | CCAGTATGGTTGTCCTCCAAA | CTGATACTGATACGGGTACTGATG | 126 | Antisense* |
|  |  | GTAGCTCATGTCTTGTGAGAGAG | CTGATACGGATACGGTTTGGAG | 139 |  |
| *BPHOGS10006054-OT-RA* | 363 | CACAGTATTCCTGTTGCGAATG | TACAGGCAGTGATGTATCCAAG | 144 | Antisense* |
|  |  | CCCGGAAATGTTTGAAAGCC | TGTGTCTTACAGGCAGTGATG | 113 |  |
| *BPHLINC250-RA* | 384 | ATGAAGGTGTTCCTACTTGTACTC | CCTATCTGTTGCGGAATTGTTTC | 200 | antisense* |
|  |  | CTCAACCAGACAACATGAAGGT | GCGGAATTGTTTCATTGTAGCC | 204 |  |
| *BPHOGS10022296-OT-RA* | 255 | GGTGTGTTGGAGGTCACTTA | GTCTCAGTTAGTGTAGCTCTGC | 204 | sense* |
|  |  | CCGTGTAGGTGTCTACAACAAA | GCAGTAGTGTCATGTACTGTGT | 228 |  |
| *BPHLNC-unc280-RA* | 319 | TGATCATCATTAGGCCTCTTTCC | GTACTGAACCACTGTCCAAGAC | 207 | antisense* |
|  |  | GCCTCTTTCCAAGGAGTTGT | TGCCAAGTACTGAACCACTG | 200 |  |
| *BPHLINC164-RA* | 379 | GGAGAGGTAATGCACCTACTGATA | ATAACGCGCTTAAAGGCAGAG | 207 | antisense* |
|  |  | CCGTGATAAGAACTTGGGAGAG | GTGGCTTATTGGAGTAGGTGATAA | 201 |  |
| *BPHOGS10026274-IT-RA* | 3490 | TTGCGTTCGCCAGAGTTT | GGATTGGTGACTCGGTCATTAG | 229 | sense* |
|  |  | GAGAGAGGACAAGACACAGAGA | GCCCAAATAGCATATCCCTAGAC | 333 |  |
| *BPHOGS10006052-AS-RA* | 370 | GAGAGCAGGCATGAACTATGAA | TTGATAACTGGACTGGCGATG | 285 | sense* |
|  |  | CTGTTGGTATTCGAATGCAGTG | CAAGGTCGGGCATAGTCATAG | 218 |  |
| *BPHOGS10017161-OT-RA* | 1005 | ATGCGTATGGCTACAGTTCG | AGACAGAGCGAGAGTGAGAA | 290 | antisense* |
|  |  | GTCTCGTCTGTAGATGGTGATTT | CGAAATGATGGCAGAATTCTAGTG | 622 |  |
| *BPHOGS10027736-OT-RB* | 1932 | ACGACGCTTCTCTTCCTCTAT | GGGCTCACTTGCTAAACTACTC | 327 | antisense* |
|  |  | CCAGGCAGTCTGTCAATCTT | CCACCAACAGGAGGAACTAAA | 256 |  |
| *BPHOGS10003291-OT-RA* | 622 | GAATCTGTGATACGTTGGCTTTG | TTATCAGGTGTGTGGGAGTAATG | 371 | sense* |
|  |  | GTTGCGTAGTTAGTGTTGTTCAG | AGCTTCAGTGATGCGTATGT | 159 |  |
| *BPHLNC-unc005-RA* | 1026 | CTAGCATCTCCCAGTTTCTCTTT | CGTTTCCTGGTTCAATCCATTTC | 377 | antisense* |
|  |  | TGGATTGGGCATCTCATTATCC | TCAACTGCTTGATCTCGAACAT | 722 |  |
| *BPHLNC-unc536-RA* | 549 | TTACCGTGATACACCTTGCTG | GCGCCAGAAGTACAGTTGATA | 401 | --- |
|  |  | CTCCTTCACTGCAATTTAGCC | CACCCTTTAGGCAAACCAATAG | 201 |  |
| *BPHOGS10000919-OT-RA* | 1613 | GCGTCTCACCAACAGATTCA | CGTATAGCGCGTGGCTTTAT | 496 | antisense* |
|  |  | GAAGAAGTGCTGAGTGCTACA | CGGCACCTAGGTATTCGATAAA | 235 |  |
| *BPHOGS10035448-AS-RA* | 719 | TGGAGCTCACTTTACAGGATTT | TTGGTGTTGCTGCCTCTT | 508 | --- |
|  |  | CAACATTGCAAAGCTGGAGAG | TGTTGCTGCCTCTTCCTTATC | 602 |  |
| *BPHOGS10030139-OT-RA* | 2435 | TGACACTAATGAAGCGGTGAA | AGGGTATTGGTACTGGATGTAATG | 565 | antisense* |
|  |  | ACTTCGGCATCGTTGAGATT | GAGGACCGACAACTCATGTTAT | 1667 |  |
| *BPHLNC-unc525-RA* | 1705 | TGAGGGTAAGGGTTTGTTCTTC | ACTGGTAGGAGTCCAGCTTTA | 1146 | --- |
|  |  | TCATGTTCGCGGAGTGTAAG | AGTCCTGTGCCGTCATTTAG | 574 |  |
| *BPHLINC406-RA* | 556 | CGGTGTCAGGTTGCTTACTT | CAGAAGCTGGTGTGTAGAAGAG | 445 | --- |
|  |  | ATCGGTGTCTGGTTGCTTAC | TGAGGGTTTCCTCTCCTTATCT | 191 |  |

*used for strand specific RT-PC
