# Supplementary material for: Genome-wide identification of long noncoding RNA genes and their potential association with fecundity and virulence in rice brown planthopper, Nilaparvata lugens
Source: BMC Genomics. 2015 Oct 5;16:749. doi: 10.1186/s12864-015-1953-y (PMC4594746; doi:10.1186/s12864-015-1953-y)
Supplement: Additional file 15: Table S11. — Primers used for RT-PCR validation of alternatively spliced isoforms of BPHLNC-unc241. (DOCX 13 kb) [file 12864_2015_1953_MOESM15_ESM.docx]

**Table S11 PCR Primers of alternative spliced isoforms of *BPHLNC-unc241***

| lncRNA genes | Forward primers (5’-3’) | Reverse primers (5’-3’) | Product size (bp) |
| --- | --- | --- | --- |
| *BPHLNC-unc241-RE* | GTACATAGGTCTGCCGAGTAAAG | GAATAATAATGGATGGAT | 772 |
| *BPHLNC-unc241-RB* | GTACATAGGTCTGCCGAGTAAAG | CTAGAAGAGTGTTTACTA | 792 |
| *BPHLNC-unc241-RI* | ACAGTGAATTACATCCAAAA | AACTGCAAATGCGTTTAT | 715 |
| *BPHLNC-unc241-RA* | GTACATAGGTCTGCCGAGTAAAG | AAGTTGTCTGCGCTAGAAGAG | 677 |
| *BPHLNC-unc241-RJ* | GTACATAGGTCTGCCGAGTAAAG | AAGTTGTCTGCGCTAGAAGAG | 867 |
| *BPHLNC-unc241-RH* | TCAGTCCATACATCCCCTAA | AGCTGTGCTCGAAAAGAG | 921 |
| *BPHLNC-unc241-RD* | CTCTTTTCGAGCACAGCT | GCATTACGATTTGGTTTT | 857 |
| *BPHLNC-unc241-RF* | ATATTAAACGCATTTCTAGC | ACCCCAATGTAACTGATG | 756 |
| *BPHLNC-unc241-RG* | TCTTCTAGCGCAGACAACTTAC | GTGGTGTGATACCGTCCATTTA | 324 |
| *BPHLNC-unc241-RC* | CTCTTCTAGAGCAGACAACTTACTATC | GTGGTGTGATACCGTCCATTTA | 339 |
| *β-Actin* | TGCGTGACATCAAGGAGAAGC | CCATACCCAAGAAGGAAGGCT | 183 |
