# Supplementary material for: Genome-wide identification of long noncoding RNA genes and their potential association with fecundity and virulence in rice brown planthopper, Nilaparvata lugens
Source: BMC Genomics. 2015 Oct 5;16:749. doi: 10.1186/s12864-015-1953-y (PMC4594746; doi:10.1186/s12864-015-1953-y)
Supplement: Additional file 16: Table S12. — Primers used for RT-PCR validation of three lncRNA genes and their overlapping protein coding genes. (DOCX 12 kb) [file 12864_2015_1953_MOESM16_ESM.docx]

**Table S12 PCR Primers of three protein coding genes and their overlapped lncRNA genes**

| lncRNA genes | Forward primers (5’-3’) | Reverse primers(5’-3’) | product size (bp) |
| --- | --- | --- | --- |
| *BPHOGS10005591* | GTACAAGCACCCTCAACTTCT | ATTCTCACCCACCCTCAAATC | 623 |
| *BPHOGS10005591-OT2* | GGCGATAAGGTAACTCCTTGTATG | GACCAGAGCCTTCAACTTGTAT | 378 |
| *BPHOGS10007976* | GCCTTGGTTGGTAGCTGTAT | CAGGCCGGACGTATGTTATT | 321 |
| *BPHOGS10007976-OT* | ATCTCACGAAGTGGTTCTTCAC | CGAAGTCGACCACAGACAAATA | 591 |
| *BPHOGS10035598* | GACTCCATGCTCGAAGAGATTC | GACGACAGAAGACCAGCTTTAT | 895 |
| *BPHOGS10035598-OT* | TCCATGACATTAGGATAACACTTCA | CTCAAAGTTTCCAAGTCCCTTAAC | 260 |
